# Movement Pattern Variability in Stone Knapping: Implications for the Development of Percussive Traditions

**DOI:** 10.1371/journal.pone.0113567

**Published:** 2014-11-26

**Authors:** Robert Rein, Tetsushi Nonaka, Blandine Bril

**Affiliations:** 1 Institute of Health Promotion and Clinical Movement Science, German Sport University Cologne, Cologne, Germany; 2 Graduate School of Human Development and Environment, Kobe University, Hyogo, Japan; 3 Groupe de Recherche Apprentissage et Contexte, École des Hautes Études en Sciences Sociales, Paris, France; University of Oxford, United Kingdom

## Abstract

The earliest direct evidence for tool-use by our ancestors are 2.6 million year old stone tools from Africa. These earliest artifacts show that, already, early hominins had developed the required advanced movement skills and cognitive capacities to manufacture stone tools. Currently, it is not well understood, however, which specific movement skills are required for successful stone knapping and accordingly it is unknown how these skills emerged during early hominin evolution. In particular, it is not clear which striking movements are indicative of skilled performance, how striking movement patterns vary with task and environmental constraints, and how movement patterns are passed on within social groups. The present study addresses these questions by investigating striking movement patterns and striking variability in 18 modern stone knappers (nine experienced and nine novices). The results suggest that no single movement pattern characterizes successful stone knapping. Participants showed large inter-individual movement variability of the elementary knapping action irrespective of knapping experience and knapping performance. Changes in task- and environmental constraints led knappers to adapt their elementary striking actions using a combination of individual and common strategies. Investigation of striking pattern similarities within social groups showed only partial overlap of striking patterns across related individuals. The results therefore suggest that striking movement patterns in modern stone knappers are largely specific to the individual and movement variability is not indicative of knapping performance. The implications of these results for the development of percussive traditions are discussed.

## Introduction

The first direct evidence for early hominin tool-use is dated to around 2.6 million years before present and consists of stone tools belonging to the Oldowan technological complex [1]. The emergence of these first stone tools represents a key development during the evolution of our species [2]–[5] and relied on the development of specific cognitive and motor skills [3] [6]–[8]. Investigations of the motor skills underlying stone knapping indicate that already these first stone tool makers exhibited motor skills well beyond the capabilities of extant great apes and even modern novice stone knappers [9]–[11]. Currently, it is not well understood how these advanced motor skills emerged during hominin evolution [12], were maintained across generations, and adapted to external constraints [13],[14]. In the present study we want to shed further light on these issues by investigating the effects of external constraints and social relationships on action adaptation and movement patterning in stone knapping using a dynamic systems theoretical framework.

### A dynamical systems perspective on stone knapping

Recent studies investigating the factors underlying stone tool technologies in early hominins increasingly have recognized the necessity to study individual behavior to understand the archaeological record [15]–[20]. However, to understand individual behavior a suitable theoretical framework is required. As has been previously noted [21], a theoretical approach particular suitable is provided by behavioral models rooted in dynamical systems theory. Under this approach, inter- and intra-individual behavioral variability is not interpreted as nuisance but as a potential source for action adaptation and exploratory behavior [22]–[25]. Key to this approach is the notion that a particular action solution is not viewed as an instance of a desired optimal pattern but rather as an emergent, self-organizing entity based on the interactions between external and internal constraints. These constraints can be differentiated into organismic constraints (internal) and environmental and task constraints (external) [26]–[28]. Organismic constraints describe all properties related to the body of the actors, including physiological, biomechanical as well as cognitive characteristics. Environmental constraints include all factors external to the actors, often beyond their influence like ambient temperature or raw material availability. Finally, task constraints describe specific task goals and imposed task rules including cultural norms and implements necessary to perform the task, e.g. tools [26]. Thereby, environmental and task constraints are not mutually exclusive and their definitions depend on the specific task and its context [26]. For example, raw material availability can be regarded as a time-independent property of the environment, thus belonging to environmental constraints, or in the context of an experiment as a specific task constraint imposed by the experimenter. Under the constraint-led perspective, task solutions always depend on the specific context and are specific to the individual actor resulting in inter-individual and intra-individual behavioral variability across actors [24]–[26]. Applied to archaeological research, the constraint-led perspective offers therefore the opportunity to include such diverse phenomena like raw material quality/availability, skill level, ecological niches, and the influence of culture into a single coherent framework [21],[29],[30]. Furthermore, regarding action adaption in stone knapping, the constraint-led approach provides the necessary theoretical background to study inter-individual and intra-individual behavioral variability.

### Skill transmission in stone knapping

Traditionally, the dynamic systems approach has been used to study constraints interactions acting upon isolated actor. Recently, however this approach has been increasingly applied to study behavioral interactions between individuals, thus modeling social contexts [31]–[33]. Marcel Mauss once pointed out that people in different cultures are brought up to walk in very different ways and wrote, “there is perhaps no ‘natural way’ for the adult [34] to walk. The same picture may hold for stone knapping, which is assumed to be one of the hallmarks of our species similar to bipedal locomotion. Just as human babies are not born walking, humans are not born stone knapping. Rather, the ability to knap stone is an acquired skill that develops in an environment that includes other members of society who are skilled at stone knapping [14]. This indicates that it is impossible to separate learning to knap a stone from learning to knap a stone in the manner conducted in one's society. Therefore, although stone knapping is certainly biological, in that it is part of a acquirable repertoire of skills of the human organism, it is also social [35]–[38]. This social aspect provides the opportunity to either implicitly or explicitly arrange task and environmental constraints such to create specific scaffolds to enhance learning [14],[29],[39],[40].

Recent evidence with respect to implicit social scaffolding shows that already the mere presence of tool artefacts creates an ecological niche which enhances action acquisition in non-human primates [40],[41]. Biro et al. [42] showed that in chimpanzees nut-cracking skills spread between unrelated individuals as well as along hereditary lines through observational learning without direct teaching [42], resulting in social scaffolding. Current evidence is inconclusive whether action acquisition of instrumental actions in extant non-human primates is based on imitation or emulation [43]–[47]. Thus, it has been argued that non-human primate cultures are lacking a ratchet effect to establish truly cumulative cultures common in humans [43],[48]. Instead, novel behavioral inventions are proposed to be based on a ‘zone of latent solutions’ (ZLS) allowing individuals to (re-)invent specific behaviors without external aid [43]. Current results from stone knapping and nut-cracking experiments however indicate that stone knapping at the level of early Oldowan is beyond the capabilities of extant non-human primates [10],[11],[49], thus lies outside their ZLS. Taken together, these examples from the non-human primate literature support the view that social components could have played a role in early hominin stone knapping activities and skill acquisition [50]. Accordingly, to better understand these social influences it is necessary to study how stone knapping skills are influenced by social groups and/or are transferred between actors.

Studying stone knapping skill transmission entails the question of what is being actually transferred between individuals. Most certainly early hominins did not possess a symbolic capacity to transfer complex physical mechanisms underlying stone fracturing. Accordingly, based on the hypothesized importance of low fidelity imitation [43],[46],[48],[51],[52], one would therefore expect that in the context of specific master-apprenticeship relations or more general social group contexts, potentially the elementary knapping action is transmitted between individuals [14],[44],[53],[54]. Thus, although according to the constraint-led perspective actors develop individual action solutions, movement patterns across teacher-student relationships and within social groups should show greater similarities compared to unrelated individuals. Accordingly, movement pattern variability within social groups should be smaller compared to movement pattern variability across groups. To better understand inter-individual movement patterning variability in stone knapping however, it is necessary to understand the influence constraints exert on the elementary actions first.

### Influence of task and environmental constraints

Recently, a single-subject study investigated the influence of chert quality on the acquisition of the Levallois reduction technique and flake morphology [55]. The results showed that the knapper adapted the knapping process in response to chert quality. Nevertheless, flake attributes showed an ongoing improvement of the knapping performance despite lower quality chert used during the latter phase of the study [55]. Thus, the knapper was able to adapt his behavior to the raw material environmental constraint to maintain performance [13],[16]. In another study, the adaptation of the kinetic striking energy of the striking hand due to changes in task constraints (hammer weight and flake size) in skilled, intermediate and novice knappers was investigated. Individuals were assigned to groups according to self-reported knapping experience. The results showed, that already novice knappers adapted their striking velocities according to task instructions and accordingly exhibit a basic capability to adapt their behavior to task constraints [56],[57]. Analysis of the striking paths further showed that all skill groups increased the striking path of the hammer when striking for a larger flake. This indicates that the individuals adapted in the same manner to this change in task constraints. Together, these two examples demonstrate the importance of controlling organismic, task and environmental constraints when studying individual adaptations in skilled performance. Both these studies however, provide no information about movement patterning and movement variability with respect to the elementary striking action in stone knapping which requires a kinematic analysis of the strike.

### Arm kinematics in stone knapping

Williams, Gordon, & Richmond [58] studied arm kinematics in flint stone knapping in two novice and two intermediate skilled knapper. The result suggested that the strike is governed mainly by elbow and wrist joint movements and is based on a proximal-to-distal acceleration pattern. This has been interpreted that stone knapping is primarily governed by force constraints [58],[59]. The authors investigated neither inter-individual variation nor differences between skill levels. Recently, this study has been extended by the authors and the influence of wrist flexion-extension movements where studied in more detail by limiting wrist mobility using a cast in eight experienced knappers [60]. Results showed significant lower precision when wrist movement were restricted. Peak joint velocities timing results again suggested a proximal-to-distal pattern. With respect to smaller accuracy when striking with a immobilized wrist, it is not clear however, whether this effect would have persisted if knappers would have trained for an equal amount of years (yrs) with a cast. Comparing the two studies with respect to peak linear velocities of the metacarpal head II suggests smaller striking velocities in the second study (range: −1.59–−3.24 m/s) [60] compared to the first study (range: −2.97–−4.08) [61]. This support the previous findings by Bril et al. [56] with respect to lower striking energy in more experienced knappers, which actually contradicts the notion that force is a limiting factor in stone knapping. However, raw materials and instructions were not completely equal across studies.

Rein et al. [62], investigated the coordination strategies of the elementary striking movements in flint knapping in seven novice and five expert stone knappers. The results suggest that both skill level groups are able to minimize hammer trajectory variability during the strike by covaring joint angle trajectories. Experts displayed significantly smaller joint angle and hammer trajectory variability compared to novice knappers but maintained a base level of movement variability. In contrast to the study by Williams et al. [58], joint angles reached peak velocity at the same time which is indicative of a precision constraint [63]. Neither inter-individual differences with respect to movement patterning across individuals nor the influence of actual performance were investigated by Rein et al. [62]. Recently, Parry et al. [64] investigated the influence of skill level on joint kinematics in stone knapping in 17 participants. Four groups were established based on the actual performance during a test condition. Investigation of the kinetic striking energy showed that the least skilled group used the greatest kinetic energy and striking arm kinematics did not show any correlations between skill or striking success with striking movements [64]. These results mirror those obtained by Biryukova & Bril [65]–[67] with respect to striking kinematics in stone bead knapping. Thus, movement patterns showed large inter-individual variability supporting individual movement solutions. However, it is not clear from this study how movement patterning varies when constraints are changed and how social groupings affect movement patterning.

### Summary and hypotheses

Taken together, the current knowledge with respect to specific movement characteristics of skill performance and intra- and inter-individual variability of the elementary striking action in stone knapping is limited. There is good evidence that that inter-individual variability with respect to the kinematics of the striking arm is present but it is not known what role this variability plays during the development of stone knapping [58],[60],[62],[64],[65]. No information at all with respect to movement patterning regarding social groups is currently available in the literature. Further, it is not well understood how variation in organismic (e.g. arm length), environmental (raw material) and task constraints (hammer weight) affect movement variability and movement patterning in stone knapping.

To address these research questions we reinvestigated the data from a group of 18 knappers including complete novices as well as experienced (+5 yrs knapping) to highly skilled knappers (+20 yrs knapping). The data had been collected as part of larger project and other data from this experiment have been previously published [57],[62],[68]. Following previous studies [58],[60],[64] we investigated joint angle trajectories to study movement patterning in stone knapping. We varied task constraints by instructing the knappers to produce flakes of two different sizes using three different sized hammers from standardized flint cores. Experienced knappers were in addition asked to produce a chopper from a basalt cobble to investigate the influence of raw material (environmental constraint). One limitation of this approach is that flint and basalt cobbles are not completely comparable with respect to outer shape. Thus, shape and material hardness are somewhat conflated in this comparison which has to be taken into account when discussing the results. We regard raw material as an environmental constraint in the context of the archaeological research, as raw material poses a time independent property of the actor's environment. To study master-apprenticeship and social group effects, we investigated movement pattern clustering across participants.

Based on the previous results, we expected joint angle trajectories to show large inter-individual variations indicative of individual movement solutions [64]. Thus, we did not expect knapping skill to depend on specific knapping movements of the striking arm. We therefore also did not expect anatomical variables, like arm length, to play a significant role, neither with respect to movement patterning nor with respect to performance. However, we expected knappers to show to some extent similar adaptations to changes of task and environmental constraints [56]. Thus, individual striking patterns should be adapted in a similar manner across individuals when striking with hammer of varying mass and/or for different sized flakes. Regarding movement patterning with respect to social groups, we expected that movement patterns share greater similarities between stone knappers linked through either a master-apprenticeship or a social group relationship.

## Materials and Methods

### Participants

18 individuals agreed to participate in the study (age = 38±12 yrs, height = 1.75±0.07 m, weight = 80±8 Kg). The data had been collected as part of a larger project [57] and results from the present experiment have been previous published with respect to hand kinematics [56] and arm coordination strategies [62] which do not overlap with the present investigation. Participants gave written informed consent prior to participation and all experimental procedures were approved by the human ethics committee of the École des Hautes Études en Sciences Sociales according to the declaration of Helsinki. Experienced participants (N = 9) E1, E2, E7 and E8 had more than 20 yrs of active knapping experience whereas experience participants E3, E4, E5, E6 and E9 had actively knapped for more than 5 yrs. For the remainder of the article experienced participants (E1–E9) are characterized by at least 5 yrs of active knapping experience irrespective of actual knapping performance during the experiment. The remaining nine participants (N1–N9) were novices and underwent a single 2 h introduction course held by knapper E1. During the introduction course the instructor first provided some general information about knapping and subsequently demonstrated the striking technique thereby explaining some key concepts including exterior platform angle and striking angle. Afterwards, participants were provided with raw flint cores and hammer stones and started to knap on their own whilst the instructor was still available and provided suggestions or answered questions by the novices. E2 was the son of participant E8. All participants, except participant E8, were right-handed and were free of injuries in the upper limbs during the three months preceding the experiments. All novices participants were recruited at the Department of Archaeology at the University of Southampton (UK). Experienced participants (E3–E6, E9) were recruited at the Department of Archeology at the CNRS-University of Nanterre in Paris (France) [56].

**Figure 1:**
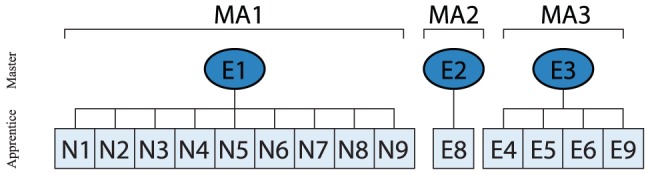
Master-apprentice and social group relationships between (E)xperienced and (N)ovice knappers, divided into master-apprenticeship groups MA1-3.

In Figure 1 the master-apprentice relationship for the studied participants are depicted. Experienced knapper E1 trained all novice knappers, whereas knapper E8 was trained by E2, and knapper E3 trained experienced knappers E4, E5, E6 and E9. Groups MA1 and MA2 where from the UK whereas and group MA3 was from France.

### Apparatus and Materials

Movements of the hand, upper arm, forearm, and shoulder of the striking arm were recorded with a electromagnetic marker system (Polhemus Liberty, Colchester, VT) at 240 Hz. Marker placement was altered if participants reported any interferences with their striking movements. Following the procedures described in Biryukova et al. [69], neutral joint positions and passive joint motions were recorded by one experimenter. Length of the upper arm and the forearm were measured from the Humeral greater tubercle to the Humeral lateral epicondyle and from the lateral Radius head to the lateral Styloid process.

### Experimental conditions

**Figure 2:**
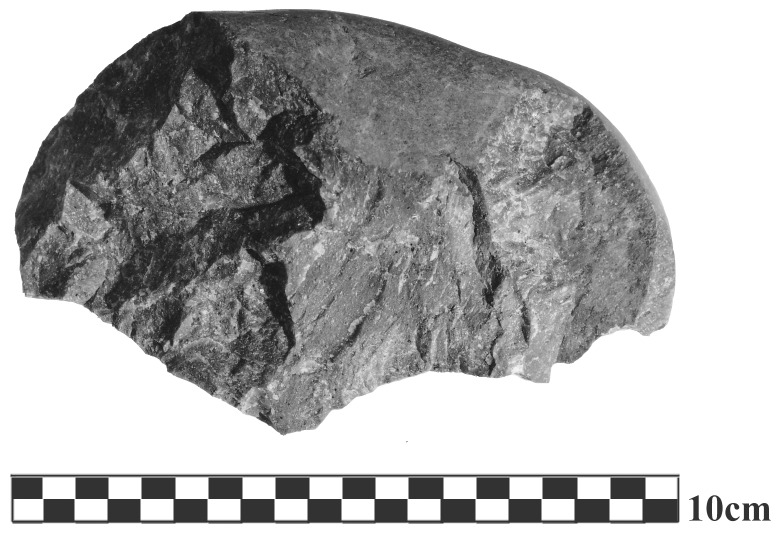
Representative example of a basalt chopper from the Oldowan condition.

Knapper E2 pre-shaped all flint cores (Norfolk flint) into a frustum (upside-down truncated pyramid, mass: 1500 g–2600 g, dimensions: approx. 130×130×120 mm), which allows continuous flaking of the side surfaces. Participants chose a preferred hammer (basalt, range presented: 420 g–680 g). Prior to each trial, participants were shown one of two different model flakes and instructed to produce a similar shaped flake (Large: 95×69 mm, Small: 52×28 mm). Each participant executed five trials for each model flake. Flake order was randomized across participants. A maximum of three strikes for each trial were allowed. Testing always started with the preferred hammer (Preferred condition). Afterwards, participants were given a 200gr heavier hammer (Heavy condition) or a 200gr lighter hammer (Light condition) and again instructed to produce three model flakes each. Order of flakes and conditions was randomized across participants. Experienced knappers were also asked to produce a chopper from a basalt cobble (Oldowan condition, always last) (see Figure 2 for an example). Knappers individually chose a new hammer and a raw basalt cobble (mass: 350 g–780 g). For participants E2 and E8 conditions Light and Heavy were not performed due to time constraints, and Oldowan data for participant E5 was lost due to a fault in the motion capture system.

Each knapper was allowed to familiarize herself with the experimental set-up by knapping prior to actual testing. All flake debris was collected and numbered. Compare Table 1 for an overview of the experimental conditions.

**Table 1:** Experimental condition matrix.

Core material	Flint		Flint		Flint		Basalt
Hammer weight	Light		Preferred		Heavy		Preferred
Instruction	Small flake	Large flake	Small flake	Large flake	Small flake	Large flake	Oldowan chopper
Experienced	x	x	x	x	x	x	x
Novice	x	x	x	x	x	x	

### Data analysis

Marker velocity and acceleration data were calculated through double finite differences differentiation using for every point x_i_ (i∈1,…, N = number of frames) the immediately preceding and following point (Δ_i_ = Δ_+_+Δ_−_, Δ_+_ = x_i+1_−x_i_, Δ_−_ = x_i_−x_i-1_, dx_i_ = Δ_i_/Δ_t_, Δ_t_ = 2/240). Using a skew oblique joint model [69], joint axis positions and angles were determined from passive joint motions and strike time-series. Elbow flexion-extension and pronation-supination, and wrist flexion-extension and radial-ulnar deviation angles were used for further analysis. All joint angle time-series data were smoothed using a second-order, zero-phase Butterworth filter with a cut-off frequency of 10 Hz. Hand marker data were visually inspected and strikes were marked using custom software written in MATLAB 8.1 (MathWorks, Natwick, MA). The beginning of each strike was determined from the first instance of positive vertical velocity prior to the maximum height of the hand marker. The instance of the impact was always clearly identifiable by a sudden inflection of the time series data as determined from the acceleration time series of the hand marker and accompanying joint angle curves. In total, 678 strikes were analyzed. Knapping skill level was judged according to knapping performance with respect to instructions instead on relying on years of training [8],[29],[55]. Following the results by Nonaka et al. [68] we assessed performance based on the square root of the summed squared differences of flake length and flake width between obtained and model flakes. Social group movement patterning relationships were investigated through striking pattern similarities using a cluster analysis approach [70],[71]. Joint angle time-series were therefore time-normalized to 100 data points and averaged for each condition (Preferred, Heavy, Light, Oldowan) and participant. Subsequently, average joint angle time series data were submitted to an average distance hierarchical agglomerative algorithm using Euclidean distances. Cluster similarity was inspected using a dendrogram.

A linear mixed-effects model was used [72] to test effects of organismic (skill and anatomy) and task constraints (condition and instruction) on joint angle kinematics of the knapping gesture at impact and joint range of motions (ROM). The independent variables were hammer weight (Preferred, Heavy and Light), instruction (Large flake, Small flake), flake success (detached vs. not detached), and anatomy. Humerus and ulnar lengths were summed to total arm length, as both variables were highly correlated, R^2^ = 0.52, t(1) = 4.2, p<0.001. To test core material effects, the same statistical model restricted to experienced knappers, preferred hammer weight, and small flakes was compared to the Oldowan condition. Only small flakes from the preferred flint condition were chosen due to their greater similarity in size to those obtained during the Oldowan condition. Statistical models were fitted using a simple random-effects structure (inter-individual intercepts) and a more complex model (inter-individual condition and instruction responses). These two models were compared with a likelihood ratio Wald-test to test for significant inter-individual differences [73]. When significant inter-individual differences were found, the fitted individual random effects were each regressed against performance scores as well as tested across groups (experienced versus novices) to investigate correlations with performance and experience.

All fitting procedures were done using restricted maximum likelihood routines using the R statistical package and lme, lme4 and multcomp routines [74]–[76]. Additional calculations were performed using custom routines programmed in MATLAB. The alpha value for all statistical tests was set to p = 0.05 and to p = 0.05/4 = 0.025 (Bonferroni correction).

## Results

**Figure 3:**
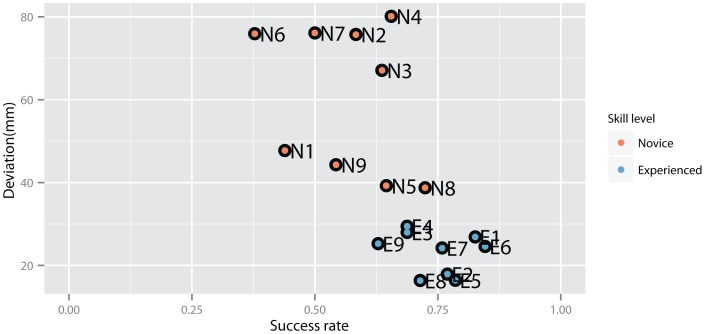
Deviation scores (differences between obtained flakes and model flakes) versus success rate (flakes per strike) for each knapper (individual knapper ids: N = novice, E = Experienced).

In Figure 3 the success rate (flakes per strike) and deviation score for each knapper are displayed. The graph suggests that in general more experienced knappers (E1–E9) have greater success rates and smaller deviations scores compared to novices. Although two novices (N5 and N8) were almost as good as experienced knapper albeit greater deviation scores. These results support the chosen approach to rate skill by actual performance instead of reported years of knapping experience. Nevertheless, statistical testing indicated significant greater success rate (Deviance = −9.8, p<0.001) and smaller deviation scores F(1, 20) = 18.93, p<0.001, for experienced knapper. Testing chosen hammer weights for experienced knappers between Preferred and Oldowan conditions suggested that experienced knappers used significantly heavier hammers when knapping harder basalt cobbles (Wilcoxon signed rank test, p<0.05). Regressing ulna and humerus lengths across all participants against deviation scores indicated no significant effects of arm lengths on performance.

**Figure 4:**
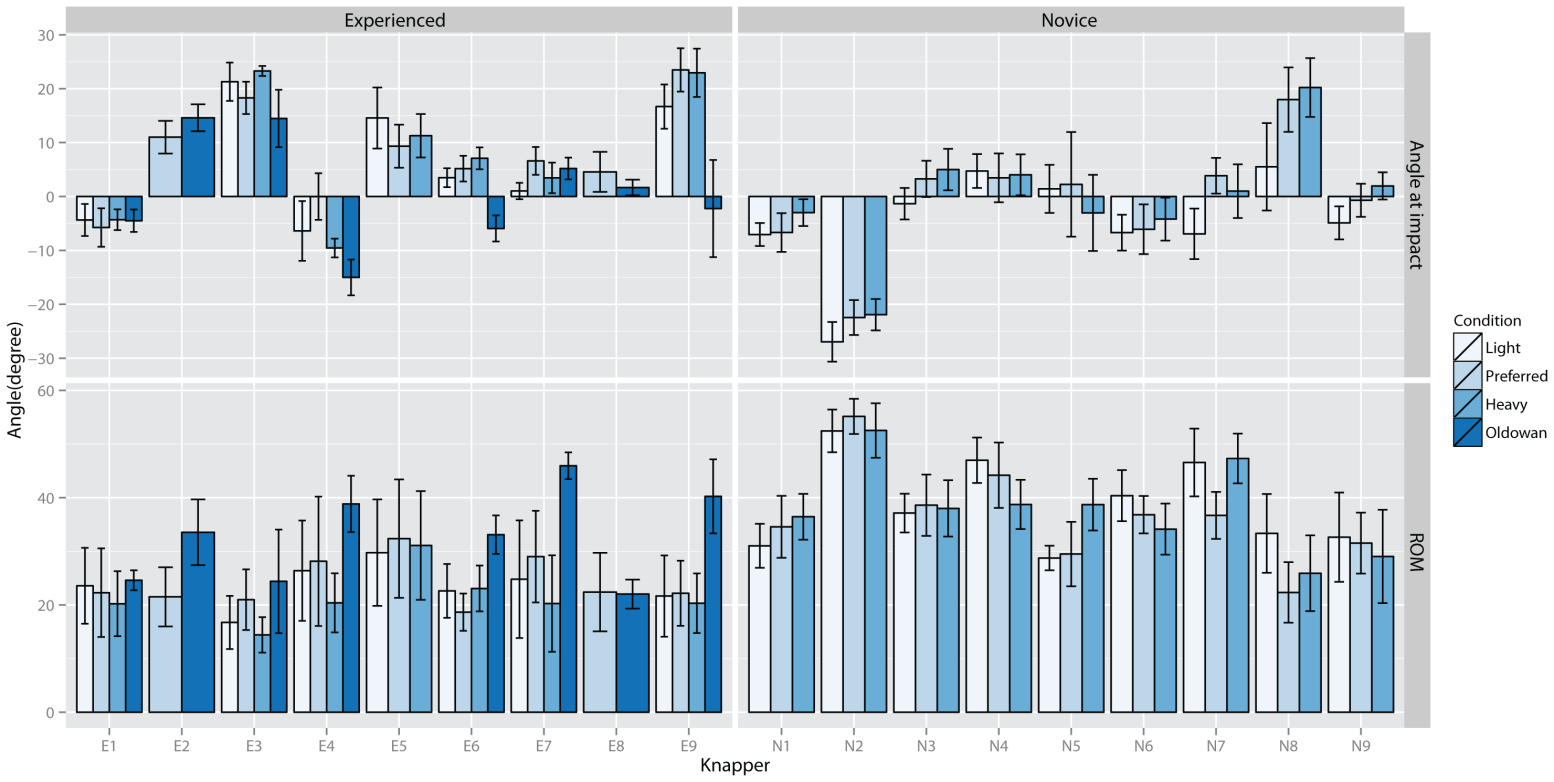
Average elbow flexion angle at impact (0° = right angle between humerus and forearm, >0° flexion, <0° extension) (top) and elbow flexion range of motion (bottom) for each knapper for each condition.

In Figure 4 the elbow flexion-extension angles at impact and according ROMs are depicted. The graph suggests large inter-individual differences of elbow joint angles at impact (Figure 4 top). Participants in both groups varied elbow joint flexion-extension impact angles around 90° and appeared to maintain the same strategy across conditions. However, knapper E9 for example, used a flexed elbow joint during all but the Oldowan condition (see also E4). Knapper N2 displayed the largest differences from the general pattern, as he struck the core with a large extension elbow angle. ROM data also indicates some distinct inter-individual variations. Experienced knappers however appear to use smaller elbow flexion angles compared to novices. Except for a trend to increase ROMs when striking a basalt cobble (all but E8), no clear trends are visible neither for novices nor experienced knappers across conditions.

Statistical testing for elbow flexion angle at impact indicated a significant effect for inter-individual differences, χ^2^(9) = 134, p<0.001. No significant group-wise main effects nor significant effects of individual differences on knapping performance or experience level were found. Thus, knappers adapted to changes in task constraints using inter-individually different strategies which had no association with knapping performance or experience levels. Testing raw material effects in experienced knappers suggested a significant random effect for inter-individual differences for conditions, χ^2^(2) = 105.9, p<0.001. No further group effects or correlations with knapping performance were found.

Testing elbow flexion ROM indicated significant effects for inter-individual responses to hammer weight and instruction, χ^2^(9) = 198.5, p<0.001, in addition to a significant group effect for instruction, F(1, 525) = 39.6, 8°±1.4°, p<0.001. Inter-individual elbow flexion-extension ROMs were significantly correlated with performance, R^2^ = 0.78, t(1) = 7.95, p<0.001, and experienced knappers used significantly smaller ROMs, t(9) = 5.2, p<0.001. Thus, larger ROMs in the elbow were associated with decreased performance although all knappers increased ROMs when striking for a larger flake (range: 1.6°–17.5°). Testing stone material effects on wrist flexion-extension ROM in experienced knappers indicated significant inter-individual effects, χ^2^(2) = 49.7, p<0.001, and significant group main effects, F(1, 176) = 37.1, 17.1°±3° SE, p<0.001. Individual responses to increased core material hardness varied between 7.8° and 26.9° and no association with performance was found. Thus, all knappers increased ROMs in reaction to increased core hardness.

**Figure 5:**
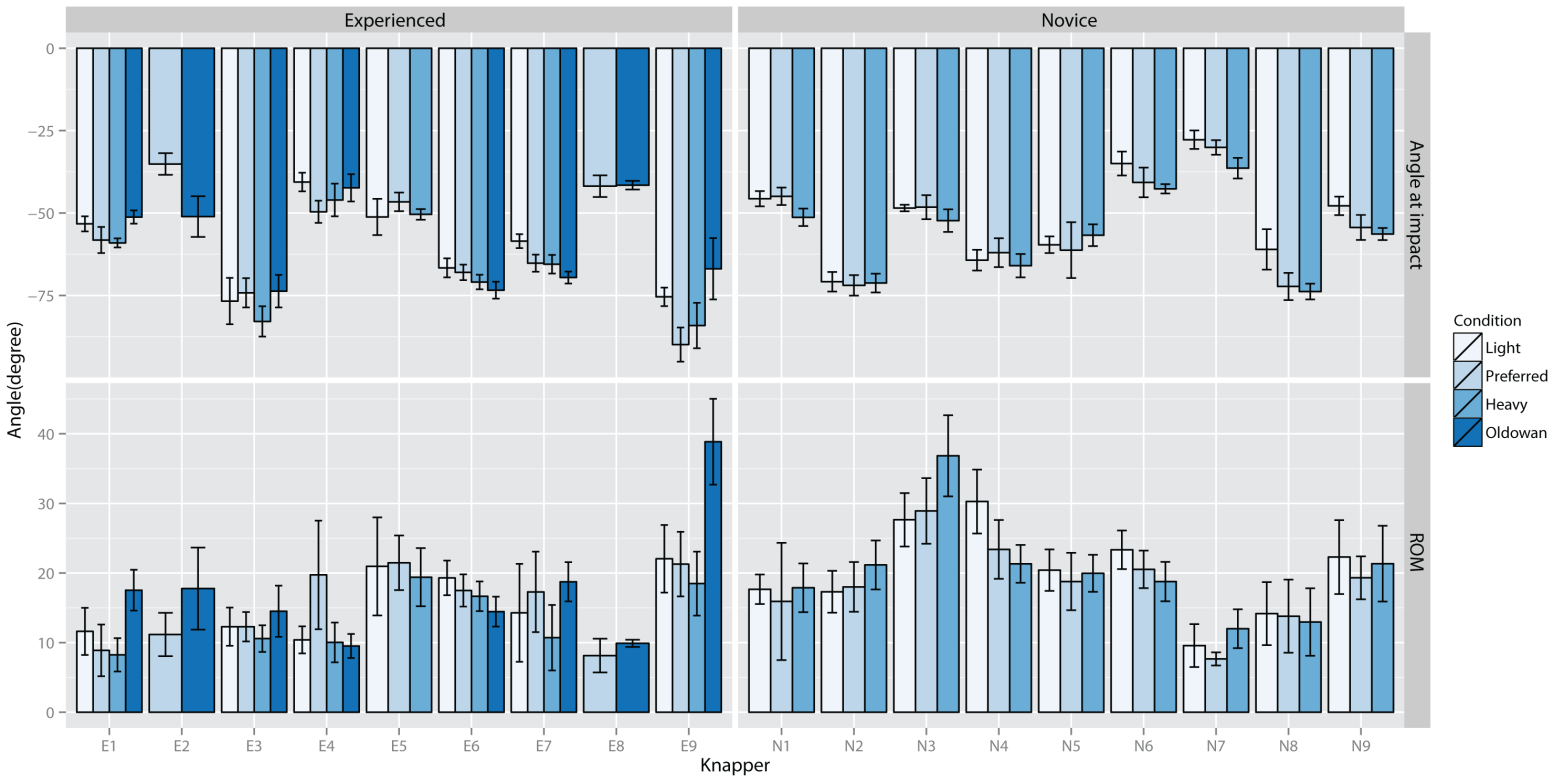
Average elbow supination-pronation angle at impact (0° = completely supinated, <0° pronation) (top) and elbow supination-pronation range of motion (bottom) for each knapper for each condition.

In Figure 5 the average elbow pronation angles at impact and ROMs are depicted. Impact postures again exhibit large inter-individual differences with no clear differences between experienced and novices knappers. However, more experienced knappers appear to use slightly smaller ROMs. Across conditions, the pronation angles appear somewhat more stable for both, angle at impact and range of motion, compared to elbow flexion angles. However, one experienced (E9) knapper increased ROM during Oldowan knapping by a much greater extent compared to the others. Thus, similar to elbow flexion angles there is no universal trend across participants and elbow pronation angles show large inter-individual variations.

At impact, statistical testing indicated significant inter-individual effects, χ^2^(9) = 159.7, p<0.001, and a significant effect for condition, F(2, 525) = 12.4, p<0.001. Post-hoc testing indicated significant differences between Heavy and Light, −5.4°±1.1° SE, p<0.001. Knappers increased elbow pronation angles when hammer weight decreased. No significant effects for inter-individual differences on performance or experience level were found. Testing raw material effects indicated significant inter-individual differences, χ^2^(2) = 116, p<0.001, but no significant group effects.

Elbow pronation-supination ROMs indicated significant inter-individual differences, χ^2^(9) = 133.1, p<0.001, and a significant group effect for instruction, F(1, 525) = 33.9, 4.3°±0.5° SE, p<0.001. Knappers increased elbow pronation rotations when striking for a larger flake. Testing of inter-individual differences indicated a significant effect of adaptation to increased hammer weight on performance, R^2^ = 0.34, t(1) = 2.9, p<0.025, as well as level, t(15) = 3.2, p<0.01, and Instruction on performance, R^2^ = 0.38, t(1) = −3.1, p<0.01, and level, t(15) = −3.3, p<0.01. Accordingly, increased ROMs in response to a heavier hammer were associated with decreased performance and more experienced knappers used smaller ROMs when striking with a heavier hammer. However, increased performance was associated with increased ROM when striking for a larger flake. Raw material testing indicated a significant effect for inter-individual differences, χ^2^(2) = 102.3, p<0.001, and condition, F(1, 176) = 5.9, 6.7°±2.7° SE, p<0.02. Experienced knappers increase elbow pronation-supination ROM when flaking harder basalt cobbles compared to flint cores. No further effects were found.

**Figure 6:**
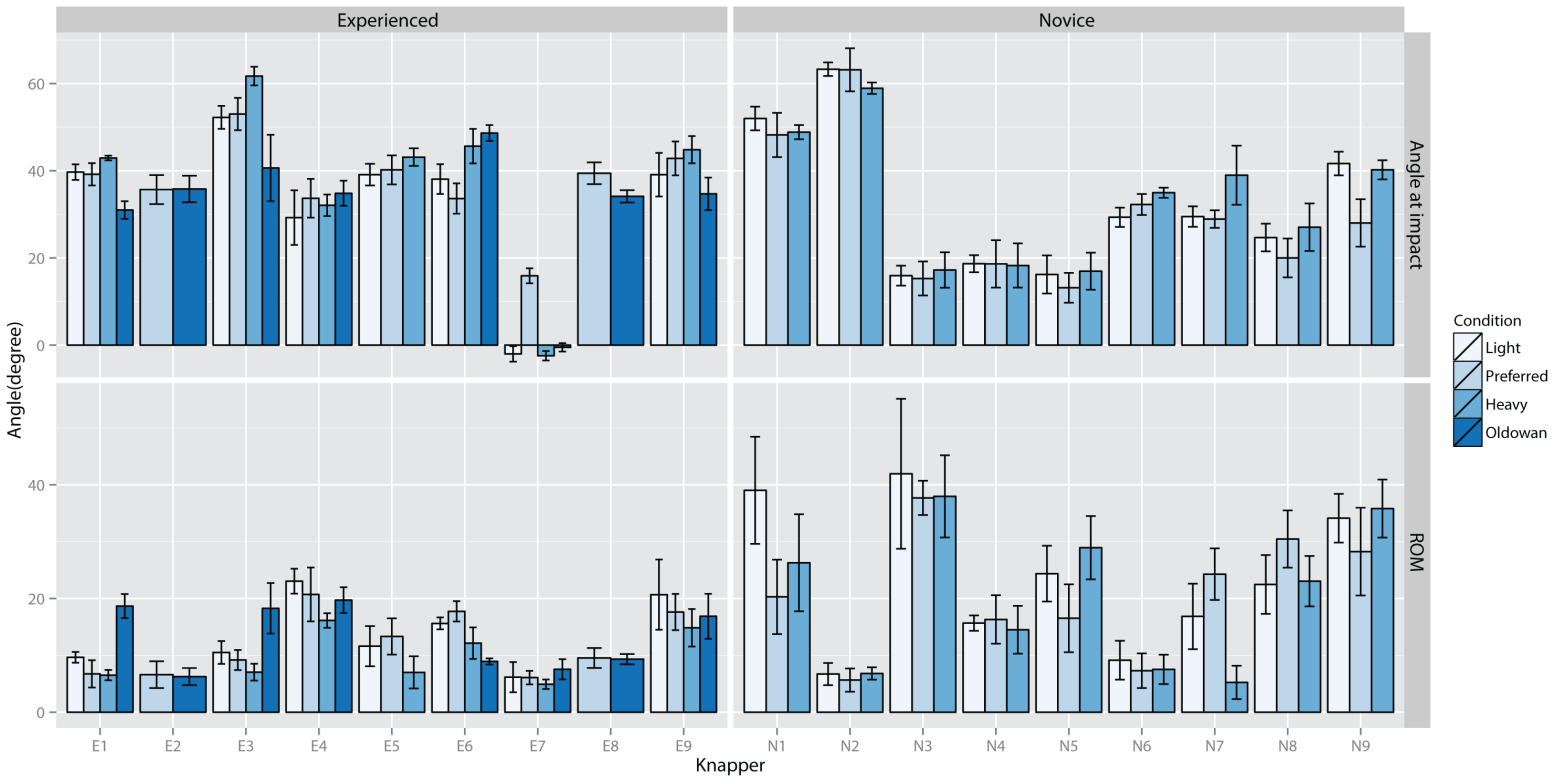
Average wrist flexion angle at impact (0° = neutral position, <0° flexion, >0° extension) (top) and wrist flexion range of motion (bottom) for each knapper for each condition.

In Figure 6 the wrist flexion-extension data are depicted. At impact, most knappers held their wrist joints in an extended position. Only knapper E7 used an almost neutral position for all but the Preferred condition. Again, large inter-individual variations are visible across participants although experts appeared to use a more similar posture with the wrist held extended at approximately 40°. ROMs appeared smaller for experienced compared to novice knappers whereas no trend with respect to differences between conditions is visible.

Statistical testing of wrist flexion-extension angles at impact suggested a significant effect for inter-individual differences, χ^2^(9) = 264, p<0.001 only. Further testing indicated a significant correlation between knapping performance and condition Large flake, R^2^ = 0.39, t(1) = −3.2, p<0.01. Improved performance was therefore associated with larger extension angles at impact when striking for a large flake. Investigating raw material effects suggested significant inter-individual effects, χ^2^(2) = 107.9, p<0.001, only.

Statistical testing for wrist flexion-extension ROM found a significant inter-individual effect, χ^2^(9) = 188.1, p<0.001, and a significant main effect for condition, F(2, 525) = 10.6, p<0.001, with significant differences between Heavy and Light, −3.4°±1.3° SE, p<0.025. No significant correlations with performance were found. Thus, knappers used smaller ROMs when striking with a heavier hammer. Testing raw material effects indicated significant inter-individual effects, χ^2^(2) = 82.5, p<0.001. No correlation of individual adaptations with performance were found.

**Figure 7:**
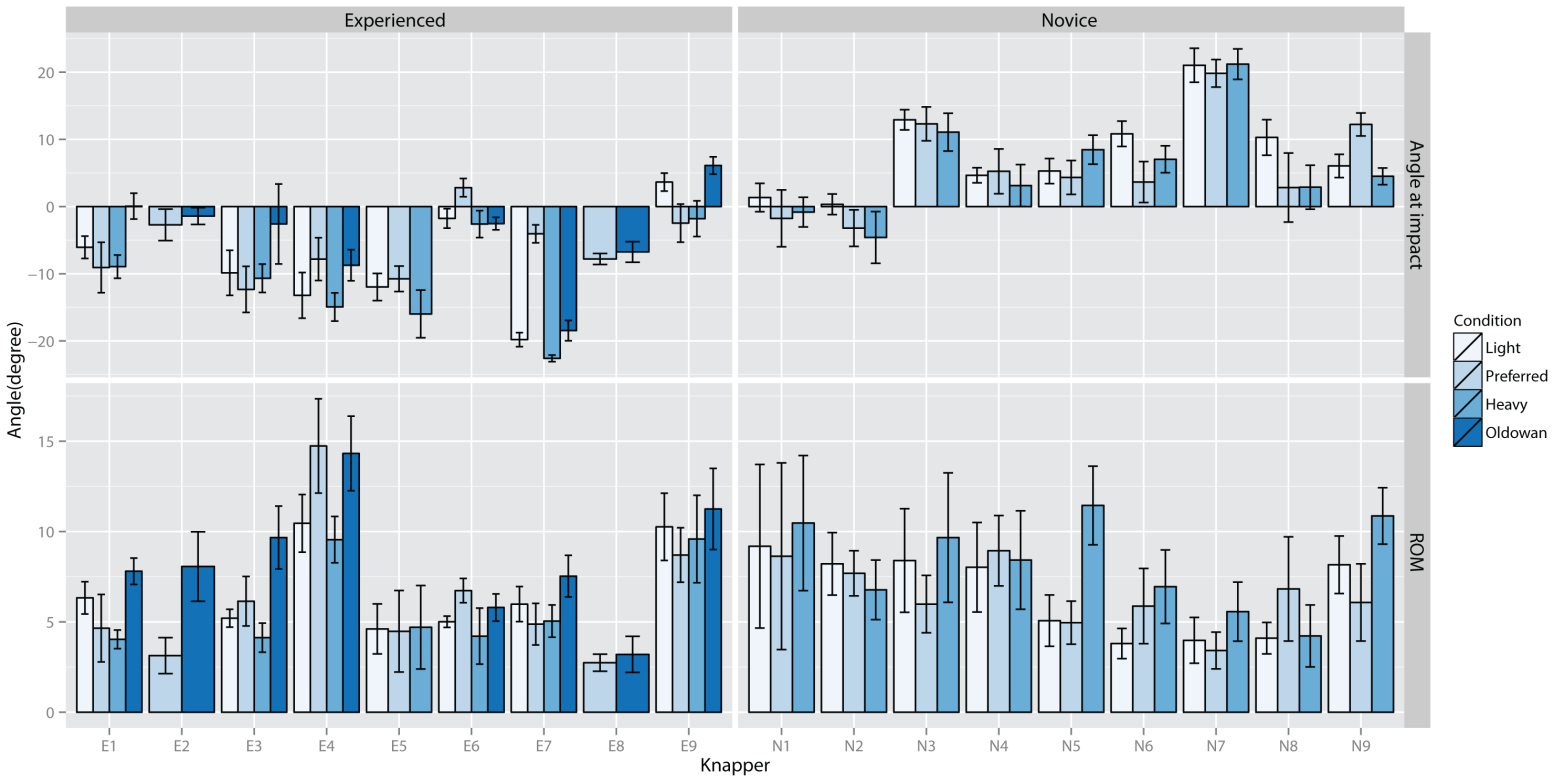
Average wrist ulna-radial deviation angle at impact (0° = neutral position, <0° radial deviation, >0° ulna deviation) (top) and wrist flexion range of motion (bottom) for each knapper for each condition.

In Figure 7 the average wrist radial-ulna deviation angles for each knapper are shown. Here a relatively clear difference between experienced and beginner knappers is visible. Experienced knappers hit the core in a radial deviation position whereas novices use an ulnar deviated position. In general, wrist ulnar-radial deviation ROMs were small (<10°). When comparing Oldowan knapping to the other conditions, there appeared to be a trend for increased ROM when striking a basalt cobble. In novices knappers, the graph suggests a trend for increased range of motion during condition Heavy, which is not apparent in the experienced group.

At impact, inter-individual differences, χ^2^(9) = 341, p<0.001, and group effects for condition, F(2, 525) = 8.2, p<0.001, were significant. Post-hoc testing indicated significant differences between Heavy and Light, −2.5°±0.7° SE, p<0.01. Knappers held their hands more radial deviated when striking with a lighter hammer. Regressing individual angles against performance indicated a significant effect for hitting angle, R^2^ = 0.39, t(1) = 3.2, p<0.01, and significant differences between novice and experienced knappers, t(15) = 4.04, p<0.001. Thus, larger ulna deviation at impact was associated with decreased performance. Further testing indicated significant effects on performance of individual adaptations to instructions, R^2^ = 0.3, t(1) = 2.62, p<0.02, and level, t(15) = 2.7, p<0.02. Better performance was associated with smaller ulnar deviation increases when striking for a larger flake. Comparison of Oldowan and Preferred conditions indicated significant inter-individual differences, χ^2^(2) = 156, p<0.001, but no significant group effects or correlations with performance.

Testing of radial-ulnar deviation ROM, indicated significant inter-individual differences, χ^2^(9) = 108.8, p<0.001, and a significant effect of individual large flake adaptations, R^2^ = 0.3, t(1) = −2.6, p<0.025, and level, t(15) = −3.2, p<0.01, on performance. Thus, increased wrist radial-ulnar deviation ROMs were associated with increased performance when striking for a larger flake. Comparing Oldowan and Preferred conditions in experienced knappers suggested significant inter-individual differences, χ^2^(2) = 20.9, p<0.001, and a significant main effect of raw material, F(1, 176) = 10.5, 2.5°±0.8° SE, p<0.01.

**Figure 8:**
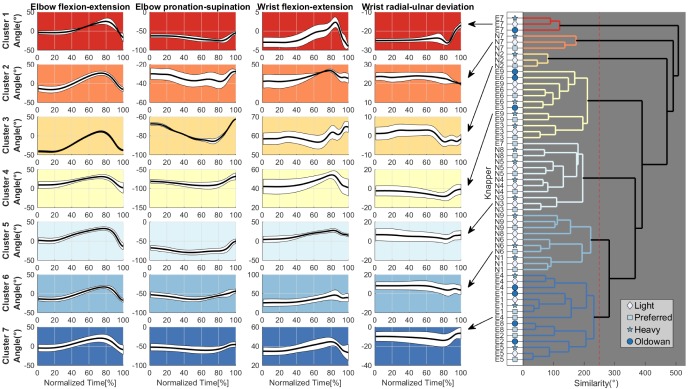
Cluster analysis results for joint angle data for Preferred, Heavy, Light and Oldowan conditions using Euclidean distances and average linkage clustering. **Clustering** of averaged joint angle data for each participant per condition (Experience: E[1–9], Novice: N[1–9]) are shown in the dendrogram (right) and average joint angles for each clusters are depicted (left plots: average trajectory ± SE).

In Figure 8 the results for the cluster analysis for the group mean data for each condition (Preferred, Heavy, Light, Oldowan) are depicted. The dendrogram shows that, except for knappers E2, E7 and N8, all movement patterns for each participant were clustered each into single primary clusters indicated by the groupings at the leftmost level. Inspecting the clustering across skill level groups further shows a clear separation into novice (Cluster 5 and 6) and experienced knapper clusters (Cluster 4 and 7). Thus, not a single cluster per level was found but rather several movement patterns for both novices and experienced knappers. Comparing the angle profiles between the two experienced clusters suggests a difference for elbow pronation-supination and wrist radial-ulnar deviation angle magnitudes. Similar visible differences exist between the absolute angle magnitudes for elbow pronation-supination and wrist flexion-extension for the novice movement patterns. Elbow flexion-extension ROMs appear somewhat greater in both novice clusters which supports the findings from the mixed-effects statistical analysis. In contrast, smaller ROMs are visible for wrist flexion-extension in clusters 5 and 6 compared to clusters 4 and 7. However, the wrist flexion-extension angles of novice Cluster 6 were of similar magnitude to those of expert Cluster 7. Similar, novice Cluster 5 and expert Cluster 4 showed similar wrist-flexion angles. Both novice clusters showed the same time profiles like experienced Cluster 7 for radial-ulnar deviations. Thus, novice movement patterns appear to be a mixture of the two experienced movement clusters. The experienced knapper E7 with no master-apprenticeship or social group relationship resided in a separate movement cluster including the Heavy, Light and Oldowan pattern whereas his Preferred movement pattern was grouped into the novice cluster 5. Using a Kruskal-Wallis test, anatomical differences between clusters did not indicate significant differences, neither for humerus (χ^2^(10) = 12.03, p>0.28) nor for forearm length (χ^2^(10) = 9.55, p>0.48). Further, investigating the relationship between the Oldowan basalt condition and the flint conditions, Figure 8 shows that the flint conditions are more closely related and are grouped earlier into a single cluster to which the Oldowan condition is latter added (compare for example E1: cluster 7).

Investigating master-apprentice and social group relationships across Figure 1 and Figure 8 shows that one of the experienced clusters contains both E2 (father) and his student E8 (son), who use highly similar joint movement patterns. This is even more remarkable as E2 is left-handed whereas E8 is right-handed and indicates that the striking technique was passed on despite differences in handedness. In contrast, the movement patterns of social group MA3 including the master-apprenticeship relationship between E3 and his students did not lead to a singular grouping. Only knappers E6 and E9 were grouped together with E3 into the same cluster whereas E4 and E5 were grouped with the other experienced knappers into Cluster 7. The results therefore suggest, that master-apprenticeship relationship do not unequivocally result in the copying of the teacher's movement patterns by the students or their social groups.

## Discussion

Currently, it is not well-understood how early hominins developed, adapted and maintained the ability to manufacture stone tools as already the earliest known artifacts indicate that their makers possessed advanced cognitive and motor skills [6],[10],[12]. Following a dynamical systems theoretical approach we addresses this question by studying the effects of social relationships and environmental and task constraints on movement patterning of the elementary striking action in modern novice and experienced stone knappers [26].

Based on previous results [56],[57],[61],[62],[68], we expected joint angle trajectories to show large inter-individual variations indicative of individual movement solutions. The present findings support this hypothesis as the magnitudes of striking angles at impact and range of motion data as well as adaptations to changes in constraints showed significant variation across knappers irrespective of experience and performance levels [64],[65],[67]. Comparing the obtained joint angle kinematics with those typically encountered in activities of daily living (ADL), shows that joint ROMs during stone knapping (average: elbow flexion-extension: 43°±15°, elbow-pronation: 27°±12°, wrist-flexion-extension: 21°±11°, wrist ulnar-radial-deviation: 10°±4°) are of similar magnitudes as during ADL, although wrist ROMs in the present study were smaller compared to normal hammering [77],[78]. However, inter-individual variability across participants appears somewhat larger in stone knapping compared to ADL [79]–[81]. Thus, the results indicate that individual movement solutions due to the interactions between organismic, environmental and task constraints should be emphasized when studying action adaptation in stone knapping [21],[26],[29],[30].

Regarding variation of task and environmental constraints we expected that on average knappers would use similar strategies to adapt to changes in constraints [56]. The results suggested significant group effects with respect to task instructions (Large vs. Small flake) for both elbow joint ROMs. Knappers across all skill levels increased elbow ROMs when instructed to produce a larger flake. For the wrist joints however, significant inter-individual effects together with the lack of significant group effects indicate that knappers did not adapt uniformly to instructed flake sizes. Results further indicated group-wide increases in pronation and radial deviation angles at strike and decreased wrist flexion-extension ROM with increasing hammer mass. This suggests that elbow and wrist joints are responsible for different aspects of action adaptation in stone knapping. However, significant adaptations in both elbow (flexion-extension ROM, pronation-supination ROM) and wrist (radial-ulnar deviation ROM) were observed when core material was changed. These findings resonate with previous findings in a ski-simulator task [82], where participants acquired a global movement pattern, which was similar across all participants but showed inter-individual variation with respect to the local coordination of limb coordination patterns [82]–[84]. The present findings with respect to variation of environmental and task constraints suggest therefore that depending on the type of change of constraints, modern stone knappers adapt their actions using either a uniform or a mixture of individual and common strategies.

With respect to task success, we found performance decrements with increased elbow flexion-extension ROMs and increased ulnar deviation at impact. The remaining effects were more specific, such that decreased performance was associated with increased elbow pronation-supination ROM when striking with a heavier hammer but decreased elbow pronation-supination ROM when striking for a larger flake. Similar, increased wrist extension angle and increased ulnar deviation were associated with decreased performance when striking for a large flake. However, increased radial-ulnar deviation ROM increased performance when striking for a large flake. Although these results support previous findings highlighting the importance of the wrist joint for successful knapping [58], they also further support the contribution of the elbow joint for successful stone knapping [64]. As has been previously pointed out [62], geometrically, variations in elbow flexion-extension movement have a greater effect on hammer movements compared to wrist movements due to the longer lever of the lower arm plus hand complex. Together with the present results this suggests differentiated joint responsibilities where both joints contribute equally to the elementary striking action in stone knapping in modern humans. This contradicts the notion that striking accuracy is influenced in a strictly proximal to distal direction [58],[64]. In general, it is difficult to assign any superior importance to a single joint along a kinematic chain as movements of any single joint always affect the movements of all other joints along the chain through interaction torques. Interaction joint torques result from inertial forces generated by joint torques. For example, joint torques generated at the elbow joint result in interactions torques affecting proximal as well as distal joints along the kinematic chain [85]. Taking further into account that many joints include muscles spanning multiple joints, it follows that simultaneous control of all joints along the arm chain is always necessary [85]–[87].

To study how knapping skill is transferred and maintained across knappers, we investigated the movement patterning in social groups including master-apprenticeships. We expected movement patterns to share greater similarities within social groups. The results provide only partial support for this hypothesis. We found two cases where social group membership resulted in high movement pattern similarity. One case represented a father-son relationship, where both knappers were grouped into the same cluster despite the master (E2) being left-handed and the apprentice right handed (E8). Potentially, as the student was exposed to the model from an early age on he was able to generalize the movement patterns from the left arm to his right arm. Investigations of nut cracking skill acquisition in extant Chimpanzees has indicated the presence of a critical age period for successful skill acquisition [42]. Potentially, a similar critical period with respect to stone knapping skill acquisition is present in modern humans which would have aided E8 during generalization of the movement patterns of his father. In the second social group, only two of four apprentices (E6 and E6) were grouped into the same movement cluster as their teacher (E3). The other two knappers (E4 and E5) were grouped with three experienced knapper (E1, E2, E8) into a second cluster of experienced knappers. This is even more remarkable as knappers E4 and E5 are from France whereas knappers E1, E2 and E8 are from the UK, yet both groups converged onto a similar movement pattern. In contrast, no clusters were found for the third social group consisting of one teacher (E1) and all novices (N1–N9). Two novices (N2 and N7) had movement patterns different from all other groups whereas the remaining novices were grouped into two novice clusters both containing more and less successful knappers (compare Figure 3 and Figure 8). Movement patterns of experienced knapper E7 differed from all other experienced knappers and his Preferred condition pattern was even grouped into one of the novice clusters. Testing for anatomical differences between clusters did not indicate significant differences. Together, these results further support the hypothesis that there is no single best movement pattern in stone knapping but rather that individual solutions are sought by the actors. As distinct groupings for experienced knappers existed however, this might indicates that successful stone knapping is supported to some extent at least by certain “more advantageous” characteristics including decreased elbow flexion-extension and increased wrist flexion-extension ROMs. Nevertheless, successful movement pattern characteristics are not hard bound as the examples of the experienced knapper E7 and novice knapper N7 (cluster 2) show. Potentially, certain movement solutions are more challenging to execute compared to others and might therefore be more preferable. For example, investigations with a skittles task have shown that different movement solutions have varying stability properties (task tolerance) with respect to external perturbations [88],[89]. Maybe some movement solutions used by experienced knappers exhibit greater task tolerance and therefore are easier to perform compared to others (e.g. Cluster 4 and 7 vs. Cluster 1). In this regard in particular the hammer trajectory could be a candidate parameter to investigate stability properties as it has been previously shown that knappers covary joint kinematics to minimize trajectory variability [62]. However, this remains an open question at present. Regarding the relation between novice and teacher movement patterns, the present results further suggest that the movement pattern similarities are not present from the very beginning of learning but require longer exposure of the teacher model until they are established. As the novice movement clusters were also more disperse as those of the experienced knappers, this might indicate that at the beginning modern novice stone knappers rather emulate the actions of their teacher and peers and rely on imitation only later on in contrast to previous results [46],[90]. However, as all learners where only trained by a single teacher the result could be due to the specific instructions given by the teacher and more research is needed.

Taken together, the results from the present study further support the suitability of the constraint-led approach to investigate motor skills in stone knapping in modern humans [21],[29],[91]. As the performance data show, the adaptations to changes in constraints are subtle and depend on the specific context. For example, in some instances an increase in elbow joint ROM was performance detrimental although all knappers increased elbow ROMs when striking for a larger flake. Movement solutions are actor specific resulting in individual adaptation patterns with respect to changes in joint angle positions and joint angle ROMs. This is also supported by the cluster analysis findings as primary cluster are constituted along individuals as opposed to conditions. Further, investigating inter-individual joint kinematic variability in four different activities of daily living (hand to contra lateral shoulder, hand to mouth drinking, combing hair, hand to back pocket) van Andel et al. [81] found relatively more consistent inter-individual movement pattern (e.g. peak wrist flexion STD 8°, peak elbow pronation STD 16°, elbow flexion STD 5°) compared to the present results (e.g. peak wrist flexion 22° STD, peak elbow pronation 26°, elbow flexion 11°). Thus, it appears that inter-individual variability in stone knapping is greater compared to those typically observed in activities of daily living [80] and highlights actor specific movement pattern solutions.

The present results further indicate however that action adaptation is not completely random across actors but does follow some common strategies. This resonates with previous findings from the sports domain [71],[82],[92],[93]. Interestingly, in these studies it has been further shown that action adaptation was accompanied by specific phenomena characteristic for self-organizing dynamical systems [94],[95]. In the study of the Levallois technique [15], the learner's performance did not follow a steady, gradual curve but performance improvement was interspersed with epochs of better performance indistinguishable from that of an expert [15]. This mirrors closely the result obtained in a soccer task, where exactly this intermittent behavior including episodes of increases performance was found too [96]–[98]. On a larger scale, this behavior mirrors also the development of stone tool technologies across the Plio-Pleistocene epochs with long periods of stasis between technological transitions, for example from Oldowan to Acheulean and latter technological complexes [50],[99],[100]. This again highlights suitability of the dynamic systems framework approach to model processes during hominin evolution. A immediate possibility to test this hypothesis could be to investigate artifact variability just before the emergence of a subsequent more advanced technological complex. According to the predictions from a dynamic systems perspective a sudden increase in artifact variability, so-called critical fluctuations, would be expected [101],[102].

Based on the present results, a tentative hypothesis can be put forward regarding the development of percussive traditions during hominin evolution. The present results demonstrate the presence of large inter-individual movement variability in modern stone knappers irrespective of knapping skill level [62],[65],[67]. Further, inter-individual movement patterns do not correlate with performance [62],[64]. This indicates that the acquisition of specific movement pattern is not necessary for successful stone knapping. In turn, this opens the possibility that inter-individual variability potentially is of functional value [27],[28]. In context of early hominin evolution this behavioral variability therefore could have provided opportunities for creative task-solution experimentation [14], and adaptive and exploratory functions [28],[103]. This would have allowed early hominin stone knappers to better adapt their technological behavior to local environmental and task constraints. Inter-individual movement variability in stone knapping within social groups thus may have provided the necessary opportunities to develop novel movement patterns more suited to local ecological niches [104]–[106]. Thereby, intra-individual movement variability could provide the background for inter-individual variability as it allows the development of inter-individual solutions in the first place. As Rein et al. [62] have shown, even experts display intra-individual movement variability at the joint kinematic level as well as at the to be controlled hammer trajectory level. Through spatial isolation and/or limited diffusion opportunities between local groups, local solutions within social groups could be consolidated and subsequently developed into local traditions [104],[107],[108].The development of local traditions based on movement pattern variability could have been supported through a stepping-stone model [109]–[111] as suggested by early Oldowan and Archeulean sites [50],[111]. Therefore inter-individually movement variability could potentially be in parts responsible for artifact variability and variability in technological practices across sites [50],[55],[112]–[115]. For example, the two experienced knappers from MA3 (see Figure 8) grouped into Cluster 7 could have formed novel groups using different movements although stemming initially from group MA3. Intra- and inter-individal movement variability therefore could have played an important role during the development of percussive traditions in early hominins. The ability to solve a particular movement problem with more than one solution should therefore not be seen as an obstacle but as an adaptive opportunity driving cultural evolution [116]. As de la Torre and Mora [17] have noted, “individual variation is real and should be taken into account because it provides the internal culture dynamic that fuels technical change” [17]. However, before further conclusions with respect to the effects inter-individual movement variability on regional differentiation the influence of raw material properties on movement kinematics and morphology variability has to be better understood.

With respect to limitations of the current study, the lack of a more systematic experimental set-up to investigate master-apprentice relationships and social group influences can be regarded as one of the main limitations. As it is not easily possible to completely control the information flow within groups and as information is most certainly exchanged not only within social groups but through interactions with individuals across social groups. Accordingly, the movement patterns found in the present study are probably also a result of information exchange across group borders through transient interactions with other individuals and social groups. Nevertheless, in all cases the social groups studied represented the main social groups of the participants and the results therefore should represent these influences. Another limitation of the present study is the fact that all novices were trained by a single expert as it limits the generalization of the findings. However, as the present knapper sample is rather larger compared to previous investigations and given the difficulties in obtaining experienced stone knappers, we are confident that the present sample served the purpose of the study.

In summary, the present study provided further support for the importance of movement variability for action adaptation in stone knapping in modern humans. The results showed large inter- and intra-individual movement variability of the elementary striking action irrespective of knapping experience and knapping performance. This rejects assumption about the presence of a singular, optimal movement pattern in stone knapping but rather indicates the existence of some more advantageous properties, which are not hard-bound however. Knappers adapted their elementary striking action according to task- and environmental constraints using a mixture of common and individual strategies. The results further showed that social group relationship are only soft-linked and actor's movement patterns are not necessarily highly similar to those of teachers or peers. These two factors, soft-linkage within social groups and large inter- and intra-individual movement variability inherent to stone knapping could therefore have aided the establishment of local percussive traditions during hominin evolution.
